# Distribution of *cylA*, *esp*, *asa1*, *hyl* and *gelE* virulence genes among clinical isolates of *Enterococcus faecium* and *Entrococcus faecalis*

**DOI:** 10.1186/1471-2334-14-S3-P32

**Published:** 2014-05-27

**Authors:** Elango Padmasini, G Divya, M Karkuzhali, R Padmaraj, S Srivani Ramesh

**Affiliations:** 1 Department of Microbiology, Dr. ALM Post Graduate Institute of Basic Medical Sciences, University of Madras, Taramani, Chennai, India; 2 Department of Paediatric Nephrology, Institute of Child Health and Government Hospital for Children, Egmore, Chennai, India

## Background

Enterococci are opportunistic pathogens causing severe urinary tract infections, surgical wound infections, bacteremia and bacterial endocarditis. Aggregation substance (*asa1*), hemolysin/ cytolysin (*cylA*), gelatinase (*gelE*), enterococcal surface protein (*esp*), hyaluronidase (*hyl*) and biofilm formation are the major virulence determinants responsible for pathogenicity of enterococci. This study was undertaken to determine virulence factors among clinical *Enterococcus* species by phenotypic and molecular methods.

## Methods

A total of 157 enterococcal isolates obtained from various tertiary care hospitals were characterized by standard biochemical methods. Virulence factors such as gelatinase, cytolysin production by plate method and biofilm formation on 96-well microtitre plate were performed for all the isolates. The presence of *cylA*, *esp*, *asa1*, *hyl* and *gelE* genes specific for virulence were analyzed by multiplex PCR with appropriate primers and cycling conditions.

## Results

*Enterococcus faecium* (84/157) was the predominant species obtained, followed by *E. faecalis* (73/157). 72/157(45.85%) strains were positive for hemolysin, 61/157(38.85%) gelatinase, 16/157(10.19%) strong and 66/157(42%) were moderate biofilm producers phenotypically. PCR results showed, 37/157(23.56%) *cylA*, 81(51.59%) *gelE*, 10(6.36%) *hyl*, 87(55.41%) *asa1* and 78(49.68%) isolates were positive for *esp* genes. Only 25/72, 32/61, 6/16 and 7/16 isolates were phenotypically and genotypically positive for *cylA* +hemolysin, *gelE* +gelatinase, *esp* +biofilm and *asa1* +biofilm, respectively. Interestingly, *E. faecalis* carried multiple virulent genes (>4 genes) when compared with *E. faecium* among our study isolates.

## Conclusion

Hemolysin and gelatinase were the predominant virulence factors expressed phenotypically whereas, *asa1*>*gelE*>*esp* were the predominant genes observed. Majority of *E. faecalis* isolates were strong and *E. faecium* were moderate biofilm producers.

